# Photoantimicrobial and Photoantiviral Textiles: Underestimated Potential

**DOI:** 10.3390/ph17091164

**Published:** 2024-09-03

**Authors:** Alexander Efimov, Serge Mordon

**Affiliations:** 1 Faculty of Engineering and Natural Sciences, Tampere University, 33720 Tampere, Finland; 2 Hemerion Therapeutics, 59650 Villeneuve d’Ascq, France; srm@hemerion.com

**Keywords:** photodynamic, antimicrobial, antiviral, antifungal, light-activated, textile

## Abstract

In this review, we summarize the present state of a rapidly developing field of light-activated antimicrobial textiles and their underestimated potential and opportunities.

## 1. Introduction

Photoantimicrobial textiles (PATXs) are closely related to the fields of photodynamic antimicrobial chemotherapy (PACT) and antimicrobial textiles in general [1]. While the latter two are established fields with over a century’s worth of history, numerous patents, and well-defined ISO standards, PATXs somehow remained in the shadow until very recently, so reviews on the topic are rare [2]. Nevertheless, we see the great potential of PATX to combat the three critical problems of modern protection against pathogens, namely (a) growing antibiotic resistance, (b) reuse of protection barriers, and (c) protection against unknown/unidentified species. In this review, we summarize the state of the art and propose directions of further development. As a special concern, we aim to raise awareness in the research community and to key industrial players of the advantages and potential benefits of this method.

The two essential components of PATXs are the textile fabric itself and the photosensitizer (PS). The PS molecule absorbs a quantum of light and goes into a state of a higher energy, in which it can react with a molecule of oxygen and relay the excess of energy to it. The reactive oxygen species (ROS) generated can be a singlet oxygen molecule ^1^O_2_, a radical species (OH, HO_2_, O_3_, etc.), or both [1]. Depending on the chemical nature and the photophysical properties of photosensitizer, either pathway (reaction Type I or Type II) can be preferred. The formed ROS molecules (which live from milliseconds to seconds, e.g., ^1^O_2_ in the air has a lifetime of ~2.8 s) can diffuse from the PS at a distance within micro- to millimeters and oxidize objects that they collide on their way. This antimicrobial action is based on so-called oxidative stress, where the microbes’ organelles are oxidized simultaneously in many places and through different reactions, making the action efficient and preventing the elaboration of resistance because of the diversity of the reaction pathways. Speaking figuratively, ROS action vs the effect of conventional chemodrugs is like a grenade vs a bullet. Most importantly, the ROS action is a universal process that is generally effective against all kinds of pathogens, including known, unknown, or unidentified bacteria, fungi, or viruses

When combining PACT action with textiles to produce PATXs, a few problems arise. Firstly, the antimicrobial action cannot be accumulated and spared for the future. The ROS are generated directly under light and their lifetime measures from microseconds to seconds, so the antimicrobial action is gone immediately after the light goes off. Secondly, the efficiency of ROS generation is directly related to the light intensity, so thick fabrics can only be efficient at the surface, but not any deeper inside the fiber. Thirdly, the PS molecules and the textile fibers undergo self-oxidation and decomposition under light, thus the photostability of PATXs is an issue. Finally, the bonding of the PS to the fabric might be challenging and requires specific considerations in terms of stability and the ease of the dyeing process.

## 2. Results

### 2.1. Textiles

Most of the studies were performed on cotton (cellulose) in woven, non-woven, and yarn/fiber forms. A few studies were made using polypropylene [3,4,5,6], as this is the material from which protection masks are mostly made. In some rare cases, polyamide [7,8,9], silk [10], or wool [11,12,13] have been employed.

### 2.2. Light Sources

The evaluation of light-driven antibacterial or antiviral textiles requires a well-designed illumination procedure in order to perform reproducible tests and validate the efficiency of the light-driven textile. The analysis of the literature clearly demonstrated that the parameters used for illumination were often missing. Moreover, since there is no standardized procedure, there is a huge diversity of light sources and illumination parameters, such as wavelength, irradiance, fluence, luminance, etc.

This paucity of information and the absence of a standardized illumination procedure is surprising as many organizations have established standards that address lighting design, safety, performance, mounting, and testing, as well as illumination levels. These standards are norms or requirements that establish a basis for common understanding and judgment of materials, products, or processes. Standards are an invaluable tool in industry and business because they streamline business practices and provide a level playing field for businesses to develop products and services. They are also critical to ensuring that products and services are safe for consumers and the environment.

On an international level, a standard has been fixed: EN62471 [14]. In a transversal way, this standard deals with the photobiological risks of lighting sources and especially LED devices and products using specific types of discharge lamps and LED light sources. EN 12464-1 [15] specifies the lighting requirements for humans in indoor workplaces, with all usual visual tasks considered.

First, it seems necessary to give some basic definitions. The illumination of a surface is defined as the luminous flux received by the surface per unit of area. It is represented by the symbol lx and measured in lux (or meter candle, or lumen/m^2^). However, there is another unit used to characterize the energy falling on a surface, which is irradiance in mW/cm^2^. It is possible to convert the luminous flux into irradiance. In fact, there is no single conversion factor between lx and mW/m^2^, as there is a different conversion factor for every wavelength. However, the wavelength of 555 nanometers, which is in the middle of the visible light spectrum, is usually used to perform this conversion.

The question now is whether luminous flux or irradiance should be used in order to activate the antibacterial or antiviral effect of a light-driven textile.

The standard IEC62471 [14] requires 500 lx illuminance for a normal office workplace. The conversion at 555 nm leads to an irradiance of 0.73 mW/cm^2^. Once again, it is surprising that none of the papers analyzed in this review have clearly defined the minimum amount of light required for efficient inactivation of bacteria or viruses in the textile.

Another factor that must be also considered is photobleaching of the dye impregnated in the textile fabric. Photobleaching (sometimes termed fading) is the photochemical alteration of a dye. This is caused by cleaving of covalent bonds or non-specific reactions between the dye and surrounding molecules. These irreversible modifications of covalent bonds are caused by the transition of the dye from a singlet state to the triplet state. The number of excitation cycles needed to achieve full bleaching varies.

Consequently, if the illuminance is too high the dye will be altered, and the inactivation of bacteria or viruses will be compromised. Only one study has evaluated the photobleaching of phthalocyanine dye **45** [16]. In this study, the light source was an LED white Sundesk 3. The illuminance at the surface of the textile was controlled with the lux meter Model LM 120 from Amprobe and adjusted to 36,000 lux, corresponding to a light power density of 1.6 mW/cm^2^ at 700 nm (1/3 of the intensity of bright sunlight, or ×60 times the intensity of a typical indoor light). The concentration of the remaining dye in the fabric was calculated. From the slope of the curve, the estimated bleaching time in sunlight (108,000 lux) was found to be 36 h, or 260 days under indoor light. This big difference reflected the light flux difference between natural sunlight and typical indoor light conditions.

Another concern is the control of the illuminance level. Except for four articles, this crucial and determinant parameter was not measured. However, illuminance can be easily determined. An illuminance meter (or a lux meter) measures the amount of visible light energy falling on to a defined area, using lux to express the available light as a measure of perceived brightness.

Inexpensive commercial devices are available. For example, Tang et al. measured light intensity using a light meter (EXTECH, Model # LT300) of around 1000 lux for efficient disinfection of their light-driven textile [17]. As a reference, they measured the light intensity outdoors under the sun (on 22 July 2020, in Davis, CA, USA), outdoors in the shade (on 22 July 2020, in Davis, CA, USA), in an office, and in a supermarket, with measurements of 87,000, 3000, 1000, and 600 lux, respectively.

Kim et al. performed measurements with a digital illuminance meter (Model LX1330B, Union City, CA, USA) to obtain a measurement of 16,000 lux [8]. Another solution consisted of measuring the luminous flux/unit area using a mobile phone. Cuthbert et al. used the Lux Light Meter app on an Apple iPhone [3].

Lastly, Chen et al. and Feese et al. used a NOVA II or an Orion power meter (Orphir Optronics Ltd., Jerusalem, Israel) to determine the fluence rate of light intensity (mW/cm^2^) [12,18].

Below is a synthesis of the different light sources and parameters (where available) used in the different studies published on light-driven antibacterial or antiviral textiles, where the diversity of light sources and the paucity of reported parameters can be clearly observed.

Sometimes, the light source was a commercial device; for example, the PDT light model LC122 (LumaCare, Salt Lake City, UT, USA) equipped with cold white light from a 150 W halogen light bulb with bundle of optical fibers was used by different teams. This light source delivers visible light (400–700nm). With this light source, Feese et al. used a fluence rate of 60 mW/cm^2^ for a duration of 15 or 30 min (corresponding to fluences of 54 or 108 J/cm^2^). Stanley et al. used a similar irradiance (65± 5 mW/cm^2^) and a duration of illumination varying from 5 to 60 min, corresponding to measurements from 19J/cm^2^ to 234 J/cm^2^ [19]. Since the spectrum of this light source can be reduced using a dichroic filter, Chen et al. irradiated their fabrics from 660 nm to 740 nm with an irradiance of 150 mW/cm^2^ and 30 s: 4.5 J/cm^2^ [20]. Similar to LumaCare, the Kruss fibre optic light source KL 5125 was put into action by Arenbergerova et al. [21].

The UV light UVA (365 nm) was sometimes proposed. The delivery of an irradiance of 3 mW/cm^2^ for 60 min (equivalent to a fluence dose of 10.8 J/cm^2^) has been reported by different authors [22,23,24]. For this wavelength (UVA), a dedicated room or chamber is required to activate the dye.

Kovacova [25] used blue light obtained from a blue LED lamp (LEDart s.r.o., Bratislava, Slovakia) with a wavelength of 470 nm and a power of 50 W.

Several authors decided to use green light, since the absorption of the dye was maximal in this spectrum region. This was the case in studies by Zhu et al. with 520 nm [26], Nie et al. with 532 nm, (85 ± 1 mW/cm^2^), and Wright et al. with 530 nm light (39 and 8 mW/cm^2^ at 4 cm, respectively) [27]. Lastly, Morsi et al. resorted to a 100 W mercury lamp (C-SHG1, Nikon corp., Tokio, Japan), equipped with a 540/25 nm filter (43 mW/cm^2^) [28].

Red light is also an option, as a 10 min irradiation with a light-emitting diode (LED) light 692 nm (12.5 mW/cm^2^) led to the complete eradication of a virus on medical mask fabric [29].

Most papers claimed that light sources were used to mimic the sunlight. However, the illuminance level was never determined nor provided. Consequently, other teams will be unable to reproduce the experiments. A xenon lamp was often used as solar simulator, for example: (i) xenon lamp, 500 W, 45 min, 15 cm [30]; (ii) xenon lamp, 3500 mW/cm^2^, 420–780 nm, 30 min illumination [31]; (iii) xenon lamp (500 W) equipped with a long-pass filter (λ ≥ 420 nm), 60 min, 65 ± 5 mW/cm^2^ , and a (iv) xenon arc lamp (500 W) kept at a lamp-to-sample distance of 12 cm from the bacteria-infected samples [32]. A 100 W tungsten lamp (1250 lm) was also proposed as a visible light source, with an average intensity of ~0.36 mW/cm^2^ at a distance of 20 cm from the sample [33]. Cardoso et al. placed a daylight lamp system at a distance of 15 cm from the sample; the incident light covered the entire visible spectrum (400–800 nm) obtained with an Osram lamp model (1380 lm, 110–130 V, 400 mA) [34].

One team performed an evaluation with three different light sources; a sunny day (Xenon, λ ≥ 420 nm, 60,000 lux), a cloudy day (lamp, 8000 lux), and a room light (800 lux) [35]. Two authors reported on illumination with a D65 (6500 K) light source. This light-bluish colored light source is used in color matching applications for paints, plastics, textiles, inks, automotive, and other manufactured products. It accentuates blue and subdues green and red. D65 is commonly used as a primary light source in color measurement instrumentation. However, no data were provided on light intensity, and only illumination durations of 10 min, 30 min, and 60 min were mentioned [36]. Li et al. also performed an evaluation of a D65 light source, but they only report the duration of illumination, which was 120 min [4].

In some cases, the irradiance was so high that the temperature of the textile reached 80 °C to 90 °C. Consequently, a photothermal effect (or photothermal therapy, PTT) was obtained, but not a photodynamic therapy (PDT) effect. When using a CEL-S3500/350 simulated daylight lamp (Zhongjiao Jinyuan Co., Beijing, China) to irradiate the samples with a vertical height of 15 cm, a temperature increase up to 90 °C was observed at 200 mW/cm^2^ [10,28]. This temperature increase was also reached when fabrics were irradiated with 808 nm NIR light. Using different irradiances (0.5, 1.0, and 1.5 W/cm^2^) for 10 min, Wang et al. monitored the temperature with an infrared thermal imager and obtained a temperature of 80 °C during illumination [13]. Similarly, Yu et al. observed bacterial killing efficiency and high temperatures when irradiating at light power densities below 300 mW/cm^2^ (808 nm) [37].

The duration of the illumination is not always well defined and can vary drastically from one study to another. A high-intensity white LED light capable of producing ~30,000 lx was used for 10, 100, and 1000 min to perform illumination [3]. Under 6 h of sunlight irradiation, Shivalkar observed that >90% of bacterial growth was inhibited in the presence of sulfur quantum dots [38]. Sometimes illumination is very long; for example, LED light (white light, 10 W) has been used at a vertical distance of 18 cm for 5 days without any other light sources [39].

Lastly, several authors provided very limited or no information. The only information was “white light for 30 min” [40], “limited-to-visible light irradiation” [41,42], or “under light” [11,13,42,43].

### 2.3. Molecules

Chemical substances used for the preparation of photoantimicrobial textiles were initially derived from the chemicals employed for PACT or PDT. Some modification was required to attach them to the fiber, though this was not always necessary.

#### 2.3.1. Porphyrinoids

**Figure 1:**
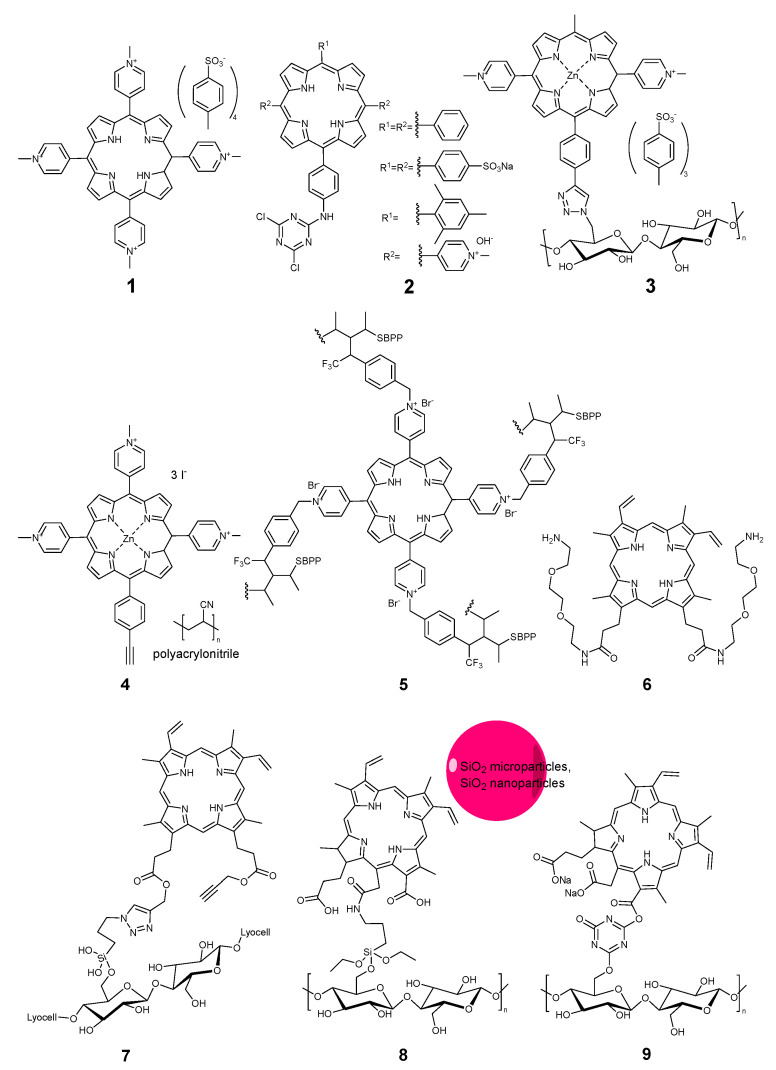
Compounds **1**–**9**.

The first mention of a light-activated antimicrobial textile can be found in the conference abstract published by Raymond Bonnett and coworkers in 1997 [44]. They reported on the modification of regenerated cellulose with tetracationic tetrakis(N-methylpyridinium)porphyrin **1** (Figure 1), without specifying which type of textile had been used. This is probably due to the relatively low stability against leaching, as porphyrin **1** was not used alone in other studies. Instead, modified compounds with an anchor group binding to the fiber were prepared and studied. Ringot and coworkers from the group Vincent Sol prepared a set of porphyrins **2**, bound to cotton via a 1,3,5-triazine link [45]. Feese and coworkers from the group Resa Ghiladi employed a different click chemistry to bind a tricationic zinc porphyrin **3** to the cellulose surface via a triazole linker [18], although they employed a paper surface, not a textile. The cationic alkylpyridinium motif gave porphyrins higher efficacy against bacteria, so this was also employed by Stanley et al. to bind porphyrin **4** to a polyacrylonitrile non-woven textile [19] and by Cuthbert et al. for the preparation of antiviral non-woven textiles made from polypropylene, to which a porphyrin photosensitizer **5** was covalently bound via diazirine linkers [3].

The development of linkers was proposed by Ringot and coworkers when they introduced protoporphyrin IX with amino-terminated linkers **6** to bind a cellulose fabric modified with polymethacrylic acid [41]. Fadavi [43] and Song [42] proposed their variations of the natural porphyrins **7** and **8** for cotton modification. The first one employed a siloxane linker and a click-triazole lock to immobilize the protoporphyrin IX, and the latter employed chlorin e6 linked via aminopropyl triethoxysilane. In addition, Song et al. embedded silica nanoparticles and coated the cotton fabric with a liquid-repellent siloxane layer. Essentially, the same chlorin structure **9**, in the form of trisodium salt (food colorant chlorophyllin), was bound to cotton via a triazine linker by Jiang and coworkers [46].

**Figure 2:**
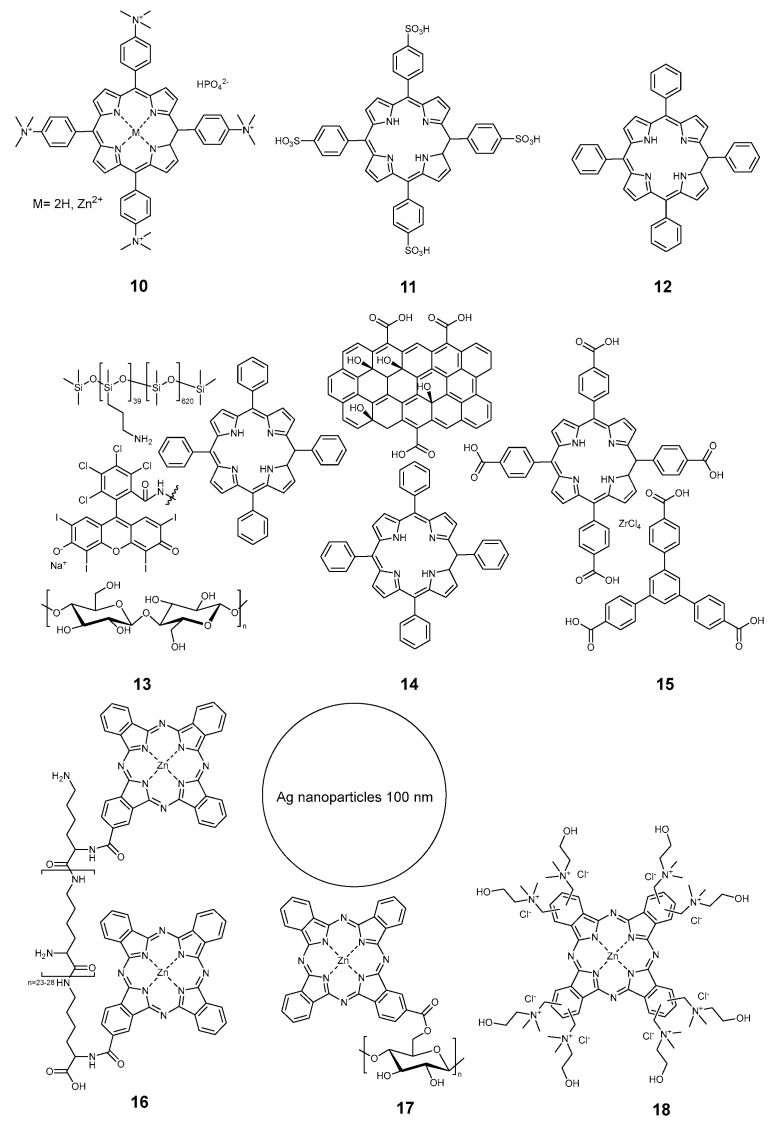
Compounds **10**–**18**.

Unanchored porphyrins were nonetheless used for the modification of textiles too. Compound **10** (Figure 2) had the cationin trialkylammonium groups used by Rahimi et al. [33]. Anionic tetrakis(p-sulfophenyl)porphyrin **11** was proposed by Castriciano and co-workers [47]. Neutral tetraphenyl porphyrin **12** was employed for dyeing textiles [21,40]. The same molecule was also used in more complex systems **13** by Wright [27] and **14** by Morsi [28]. In the work of Wright et al., the porphyrin photosensitizer was used along with polisiloxane-bonded Rose Bengal as a coating for the cotton fabric. Morsi proposed the use of graphene oxide as a co-producer of singlet oxygen over the cellulose acetate membranes. Nie and coworkers employed a rather complex structure, including graphene quantum dots and nanoparticles prepared from a tetracarboxy derivative of TPP and zirconium-based metalorganic frameworks **15** to modify preliminary aminated knitted cotton fabrics [30,48,49,50].

**Figure 3:**
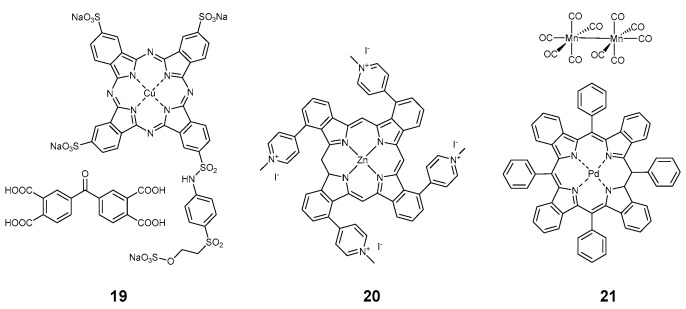
Compounds **19**–**21**.

Few phthalocyanines were also used for the preparation of photoantimicrobial textiles. In 2017, Chen et al. covalently linked monosubstituted zinc phthalocyanine to a polylysine chain **16**, and a cellulose fabric was impregnated with the obtained polymer [20]. Two years later, same group proposed a combination of covalently-bound phthalocyanine and 100 nm big silver nanoparticles **17** as a combined antibacterial agent on the cotton support [51]. Kurskaya and coworkers proposed polycationic octakis(cholinyl)zinc phthalocyanine **18** for antiviral impregnation of medical masks, and the exact material of the mask was mentioned in the article [29]. A combination of tetrasulfonated copper phthalocyanine and tetracarboxybenzophenone **19** (Figure 3) obtained from commercial sources was employed by Hu et al. to modify cotton fabric [23]. In 2024, Efimov and coworkers proposed an antiviral cotton textile based on tetracationic zinc phthalocyanine **20**, which proved to be stable and efficient [16].

All the porphyrinoids mentioned above had antimicrobial functions based on the generation of reactive oxygen species. One work stands out from this trend, as it proposed a red light-induced release of carbon monoxide CO from a sacrificial source of carbonyls sensitized by palladium tetrabenzotetraphenylpoprhyrin **21** [52]. Antimicrobial studies were not performed though, probably due to the low stability of the textile. The authors mentioned that it had to be stored and refrigerated in the dark and used within 10 days to avoid spontaneous decomposition of the dimanganese dodecacarbonyl.

#### 2.3.2. Keto and Quinone Dyes

Benzophenone and anthraquinone dyes were also proposed as photosensitizers for PACT textiles. Oh et al. employed an aminated derivative of benzophenone **22**, though the dye had to be activated by UV light [24]. The tetracarboxy benzophenone was also used a co-sensitizer for phthalocyanine, as it was mentioned earlier [23]. Larger benzophenone derivative **23** in combination with chlorogenic acid was used to photosensitize a non-woven polypropylene [4]. The light absorbance occurred chiefly at 400 nm. Very recently, Zhang et al. proposed a phosensitized wool, which was modified by benzophenone tetracarboxylic dianhydride bound to lysozyme **24** [11]. In this case the light absorbance occurred in the 400–500 nm range.

**Figure 4:**
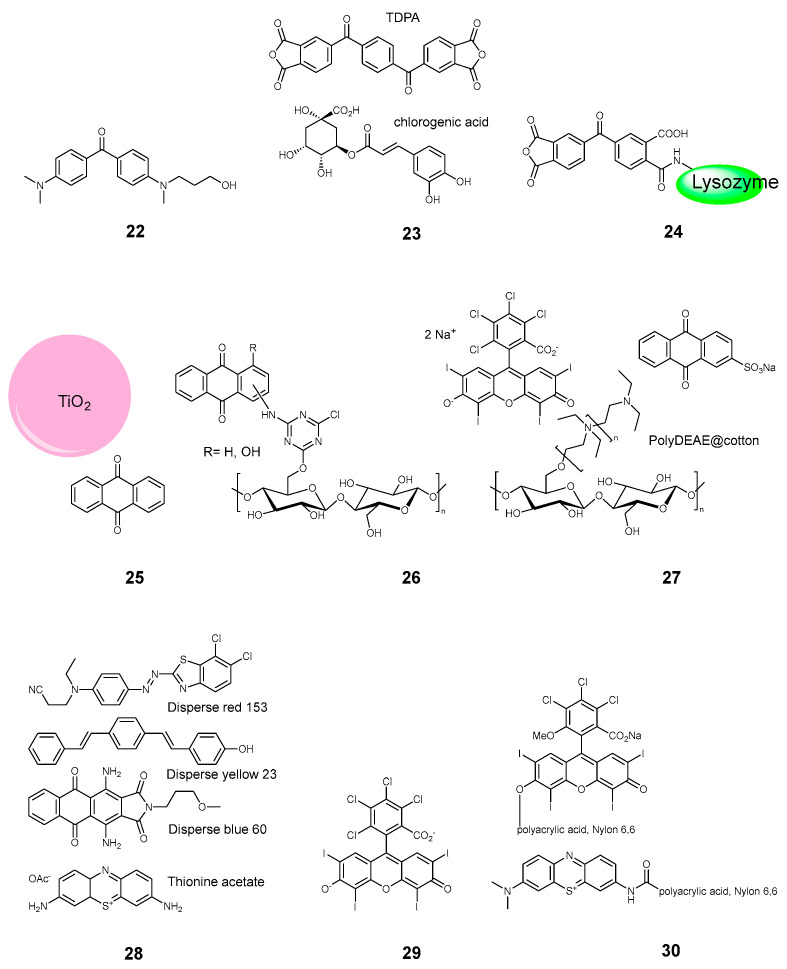
Compounds **22**–**30**.

Anthraquinones derivatives might be strongly colored in the visible range, giving red and blue shades. Rahal and coworkers employed unsubstituted anthraquinone in combination with TiO_2_ **25** (Figure 4), which absorbed the violet light at 420 nm [53]. In the work of Cardoso and coworkers, the hydroxy-substituted anthraquinone **26** was covalently bound to an unbleached cotton fabrics via triazines [34]. As a different approach, Tang et al. decorated the cotton cloth with poly(diethylaminoethyl) chains, to which a sulfo-derivative of anthraquinone **27** and Rose Bengal dye was bound electrostatically [17]. A commercial anthraquinone derivative, Disperse blue 60 **28** was used by Jiang et al. among other commercial textile dyes [39].

#### 2.3.3. Xanthene Dyes

Rose Bengal (RB) **29** and related compounds are among the most popular dyes used for research on PACT textiles. Available commercially, with a known safety profile, they are beautifully colored and relatively stable. In its unmodified form Rose Bengal was used to color the polyamide fabrics [7]. Jin and coworkers employed it in conjunction with a long phosphorescing SrAl2O4:Eu2+, Dy3+ on cotton fabrics [54]. Lee et al. used Rose Bengal in combination with petal-like nanostructures to prepare self-disinfecting polypropylene cloth [5].

**Figure 5:**
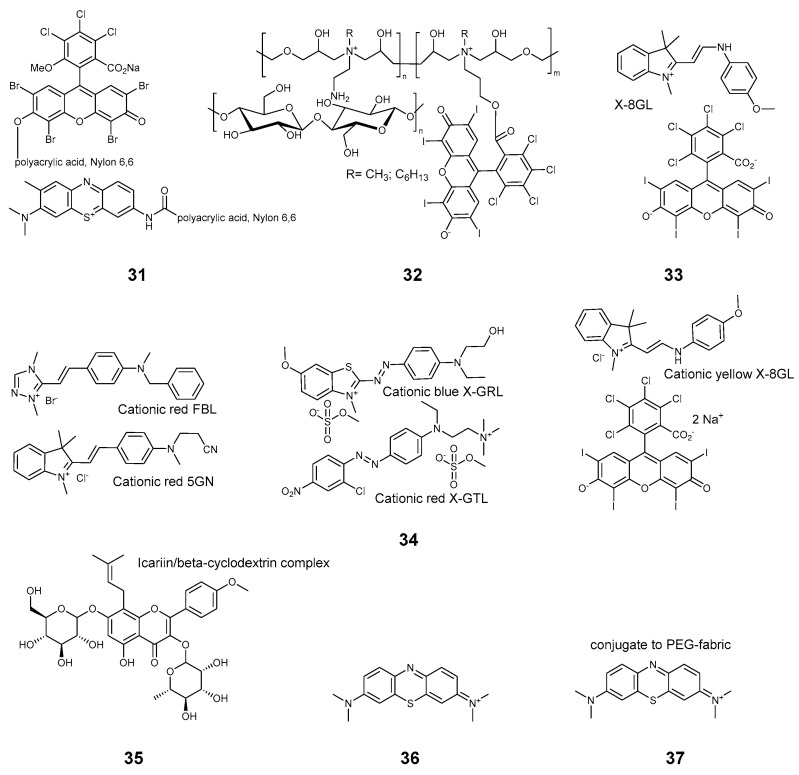
Compounds **31**–**37**.

A series of articles was published by Kim and coworkers, in which Rose Bengal and related Phloxine B **30**, **31** (Figure 5) were covalently bound to polyacrylic acid [55] and applied to nylon 6,6 [8,9] in combination with a phenothiazine derivative **30**. Covalently bonded Rose Bengal was employed by Zhu et al. in a synergistic cotton-based fabric **32**, which also included polyammonium motifs [26]. A mixture of RB with commercial dimethylindolinium dye cationic yellow X-8GL **33** was used to prepare antimicrobial wool-acrylic blended fabrics [12]. Later, an expanded set of commercial dyes **34** was tested by this group on a wool-acrylic blend [13]. It should be noted that the set of compounds **34** employed in the study were used as received from commercial sources, and labeled with their commercial names, thus the identification of the substances might be problematic.

The synergistic effects of RB, anthraquinone, and cationic polyamine were studied in the aforementioned works of Tang et al. [17], and the effects of porphyrin were studied by Wright et al. [27]. Not exactly xanthene, but a structurally related flavonoid icariin **35** was used to prepare an antimicrobial cellulose material by Mensah and coworkers [32]. Icariin dye is used in traditional Chinese and Korean medicine as an erectile function enhancer.

#### 2.3.4. Phenothiazines

Methylene blue (MB) and its related compounds are among the oldest and most proven antimicrobial substances used in medicine. Safe for humans, they are usually less light-stable than the other textile dyes. Nonetheless, numerous studies employ various xanthene derivatives. According to Kim et al., MB can be bound covalently to the polyacrilyc acid **8**, and this compound can be further employed to modify nylon cloth [8,9]. Unmodified MB **36** can be used to make antimicrobial polyester/cotton blends [31]. The effect of MB was studied on a cotton cloth in the aforementioned test of commercially available dyes [39]. A recent work by Youssef et al. proposed that polyethyleneglycol fabrics be dyed with MB **37** for wound healing [56].

#### 2.3.5. Dendrimers and Polymers

**Figure 6:**
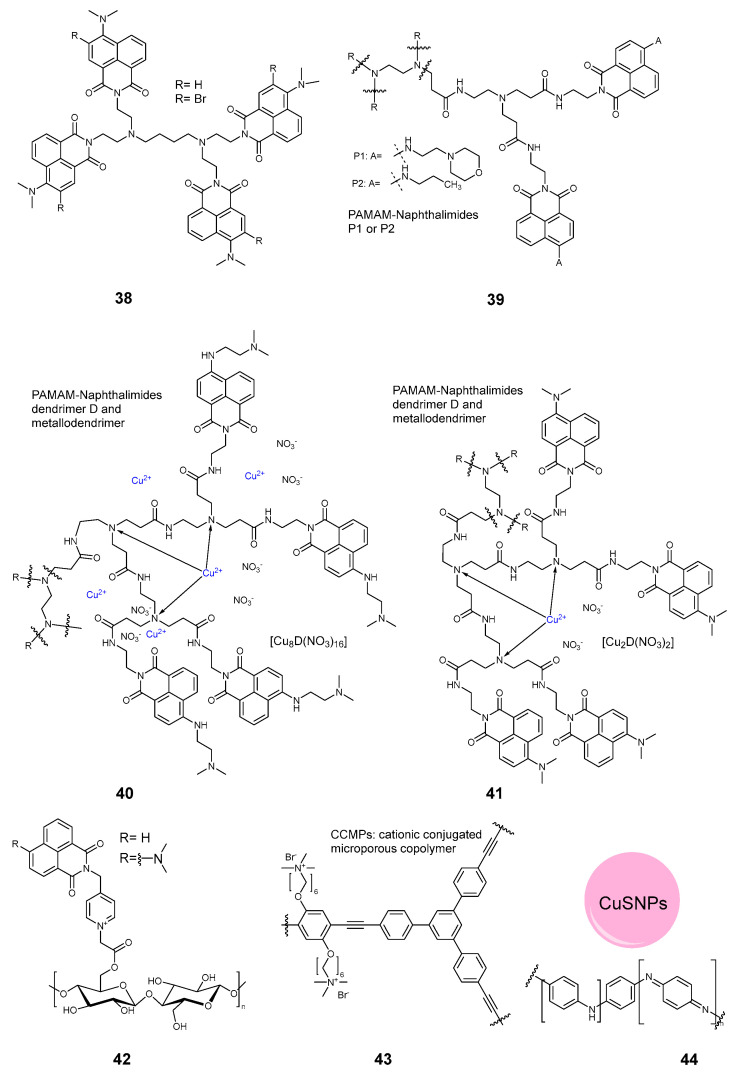
Compounds **38**–**44**.

A systematic study of cotton fabrics modified with derivatives of 1,8-naphthalimides was undertaken by the group Grabchev. Starting from a relatively small tetramer **38** (Figure 6) [57], the system was expanded to a PAMAM-napthaalimide dendrimer **39** [58]. The dendrimers were later modified with copper ions **40** [59], and the naphthalimide substitution was varied **41** [60]. Apart from the dendrimers, the naphthalimides were appended directly to cotton cloth **42** using chloroacetyl chloride chemistry [61].

As for the polymeric photoantimicrobial agents, a modification of polypropylene cloth was proposed by Wang et al., employing a cationic conjugated microporous copolymer **43** [6]. The absorbance profile of the fabric was maximal at 400–420 nm. Another amino polymer was proposed by Ren and coworkers [10]. In this work, a conjugated polyaniline **44**, in conjunction with copper sulfide nanoparticles and polydimethylsiloxane, was employed to modify silk fabrics and make them highly stable against deterioration.

#### 2.3.6. Nanoparticles and Inorganic Sensitizers

**Figure 7:**
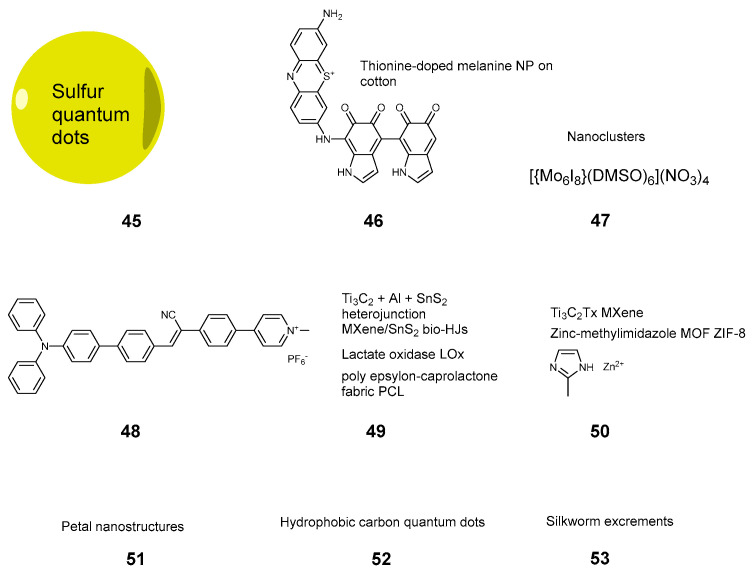
Compounds **45**–**53**.

Nanoparticles became a hot topic at the beginning of the century, as their properties significantly vary from the bulk materials. In PATXs, silica nanoparticles **8** were proposed to modify cotton cloth [42] in combination with chlorin e6. Copper sulfide nanoparticles were used in the work of Ren et al. [10] to modify silk. Sulfur quantum dots **45** (Figure 7) were employed to make cotton cloth photoantimicrobial [38], though the difference between light and dark activity was within 1 log. A rather unusual example of nanoparticles was proposed by Liu et al. [62]. In this work, methylene blue was employed to dope melanine nanoparticles on cotton **46** to exert a PACT action. Cotton fabric was used as a textile support, and the bacterial infections were cured on wounds in mice. Novikova et al. employed molybdenum-based nanoclusters **47** for photodynamic inactivation of bacteria and viruses on cotton [63].

Somewhat in relation to the nanoparticles, Li et al. employed the effect of aggregation-induced emission to create a photosensitizer from clusters of a triphenylamine derivative **48**, which was then applied to a polypropylene cloth [64].

Inorganic materials have been used for PACT textiles in the form of supramolecular structures. Wang et al. employed rather complex titanium carbide/aluminium/tin sulfide heterojunctions (“MXenes”) **49** with lactate oxidase to render the epsilon-caprolactone fabrics antimicrobial [65]. A combination of similar MXenes with a zinc-methylimidazole metalorganic framework **50** was used to modify cotton [37] and cellulosic non-woven [66] fabrics. Petal nanostructures 51 demonstrated antimicrobial activity on the surface [5]. Hydrophobic quantum dots **52** [25] and silkworm excrements **53** were also proposed to make the antimicrobial coating [35]. In the latter case, however, 70% of the pathogens were eliminated in the dark, with no light irradiation.

### 2.4. Efficacy against Bacteria, Fungi and Viruses

Antimicrobial efficacy is probably the most complex topic in this review. The results of chemical preparations, photochemical measurements, and stability tests can be obtained via established procedures and reported in a concise manner as a descriptive set of values. Although, the results of the antimicrobial tests are multiparametric by nature and depend on a great number of variables.

When evaluating the antipathogen activity, the following parameters must be controlled.

1. Type and strain of pathogens;
2. Surface-to-volume ratio between the tissue and the pathogen inoculum media;
3. The load of the dye per unit of area of the fabric;
4. The nature and intensity of the activating light;
5. The illumination time;
6. Filtering of heat;
7. Dark activity;
8. Blank/negative control;
9. Numerical assessment of inactivation;
10. Number of parallel experiments.

Ideally, a standardized method should be used in all cases. To date, few standards known for the testing of antibacterial textiles [67]. The quantitative methods are AATCC 100 and JIS L 1902—the absorption method, and the qualitative ones are AATCC 147 and JIS L 1902—the Halo methods. Antiviral textiles can be tested according to ISO 18184:2019 [68,69,70,71]. However, none of these tests were designed for photoactivated textiles, so the protocols should be modified. The modified AATCC 100 method was followed by Cardoso, Castriciano, and Chen et al. [20,34,47,51]. The modified ISO 18184:2019 was used by Efimov et al. [16]. The standard ISO 22196:2011 [72] “Measurement of antibacterial activity on plastics and other non-porous surfaces” was employed in the work of Kovacova et al. [25].

The results of the antimicrobial tests are summarized in Table 1. The numbers are given as rounded up values, taking a logarithm of the eradication of a pathogen; i.e., “6” means 99.9999% inactivation and “0.2” means 63% inactivation. Most of the experiments were performed on bacteria of a so-called ESKAPE set, chiefly *E. coli* and MRSA. Antiviral experiments were performed on the influenza virus and on variations of SARS-CoV2 (227 and 229E), as well as on non-enveloped types and other types (Table 1). As for fungi, an extensive set was tested by Kim et al. [8,9], including *A. fumigates*, *A. niger*, *Trichoderma viride*, *P. funiculosum*, *Caetonium globosum*, *P. cinnamoni*, *M. grisea*, and *C. albicans*. The last one was also employed in the work of Novikova et al. [63].

In most of the tests, the surface activity of textiles was evaluated quantitatively. That is, a small inoculum volume (100 µL) was deposited over a few mm specimen of the textile, and the pathogens were extracted after irradiation, cultivated, counted, and compared to a dark or negative control. In that sense, tests were performed similarly to the AACTT 100 standard, so the results can be directly compared. Also, in most of the works, blank experiments with no photosensitizer were performed.

Few works, however, evaluated the possibility of inactivation in volume, where a centimeter-size textile specimen was placed into a few ml of cultural liquid and the growth reduction measurements or counting of colonies were performed after illumination [7,8,9,31,60]. In one study, the activity was evaluated in a suspension of cellulose nanocrystals with inoculum [18]. Another one employed a fiber placed onto an agar surface, and the inhibition zones were estimated [43]. In these cases, the results are much more difficult to compare and can be used as semi-quantitative or qualitative indications.

For various non-textile antipathogen applications, a borderline 3-log (99.9%) reduction in CFU after treatment is a commonly accepted value to use the word “antimicrobial”. The AATCC 100 test requires a 4-log (99.99%) reduction in CFU over a 24 h contact of the textile and inoculum. In this case the textile can be called “antimicrobial”. On the other hand, the antiviral standard ISO 18184:2019 classifies a 3-log reduction as “excellent” activity.

Since a 24 h irradiation does not seem to be practical, the illumination times in the studies typically vary between 10 min and 4 h, most commonly being 30 and 60 min. Therefore the 3-log reduction in CFU could be taken as a reasonable borderline value. In this case, most of the published works faithfully claimed their textiles to be “antibacterial”. The antiviral studies can be also considered to be mostly successful [3,4,16,39]. Proper antifungal activity is much more difficult to achieve though. Based on the formal criteria, only *A. fumigatis* was successfully suppressed by photoactive textiles [55].

Photoantimicrobial efficacy depends dramatically on the incident light. The absorption profile of a photosensitizer, the irradiation light power and its density distribution, and the time of irradiation must be counted. These three critical parameters together can be expressed as an absorbed light dose in J/cm^2^. By neglecting the absorptance profile, the irradiation light can be described by the irradiated light dose in J/cm^2^.

For the sake of comparing different reports, we propose the use of the irradiated light dose. Indeed, different chromophores absorb at different wavelengths and different light sources have different emission profiles. Antimicrobial textiles, however, are expected to work under available light, be it indoors or outdoors, and the indoor light sources used in living spaces are more or less successfully mimicking sunlight. Also, accurate measurements of light intensity require special equipment, which is not easily available, whereas measurements of illuminance in lux are easy accomplishable with an affordable equipment, and the results can be easily interpreted.

We propose the use of an approximation employed nowadays for estimating the efficiency of photoelectric elements, as presented in Table 2. The power density of direct sunlight can be roughly taken as 100 mW/cm^2^, which corresponds to 100,000 lux illuminance. A bright daylight outdoor swould produce 10 mW/cm^2^ and 10,000 lux. In a shadow, the light power would be 1 mW/cm^2^ and 1000 lux illuminance, and indoors in an office the light would be considered as 0.5 mW/cm^2^ power and 500 lux illuminance. The data in the Table 1 were recalculated according to this principle. Where possible, the light power density provided in the article was used directly to calculate the light dose as

Light dose (J/cm^2^) = Light power density (mW/cm^2^) × Illumination time (s) × 10^−3^

As in most articles, the light power density was not given, so it was estimated from the illuminance as

Light power density (mW/cm^2^) = Illuminance (lux) × 10^−3^

Using these equations one can calculate that 30 min of sunshine (100 Klux) would give a 180 J/cm^2^ light dose, 30 min on an overcast day (10 Klux) would be 18 J/cm^2^, 30 min in a shadow (1 Klux) would be 1.8 J/cm^2^, and 30 min under office lighting (0.5 Klux) would give 0.8 J/cm^2^. Hence, one could grade all the reported experiments into four categories by the irradiated light dose, so the required illumination conditions for efficient antimicrobial function would be

- Direct sunlight, 100–1000 J/cm^2^;
- Bright day, 10–100 J/cm^2^;
- Shadow outdoor, 1–10 J/cm^2^;
- Indoor, 0.1–1 J/cm^2^.

By employing this classification, one can see that only compounds **3** and **20** are suitable for indoor use with acceptable efficiency. The dyes are tetracationic porhyrinoids with alkylpyridinium substituents. On the other hand, a tetracationic porphyrin with alkylammonium substituents **10**,showed weaker performance.

Compounds **2**, **6**, **10**, **11**, **29,** and **48** can be classified as suitable for use in a shadow. These are either porphyrin derivatives or alkylpyridium compounds, which match well with the observations for the most efficient group. Rose Bengal **29** [7] also can be quite an efficient photosensitizer, although other sources reported much higher light doses employed for activation [8,9,12,54,65]. Other compounds, including quantum dots and industrial dyes, require bright or direct sunlight to be sufficiently active.

Photoinactivation of bacteria and viruses can be quite successfully accomplished. Unfortunately, this is not true for fungi. The reported studies [8,9,63] demonstrate that there is still a lot of work to be undertaken.

### 2.5. Dye Load and Stability Tests

Apart from the light dosage, the amount of the photosensitizer also plays a crucial role in the overall efficiency of the material. The mechanical stability of colored textiles, their light fastness, and washing and rub fastness are also crucially important from a practical point of view. These parameters are summarized in Table 3.

Comparison of the dye loads could give a rough idea about the relative efficacy of different photosensitizers. Unfortunately, the provided data are difficult to compare. In many articles, no dye load values are given at all. In others, they were given either as a mass % of the material, mol per gram of the fabric, or mol or gram per area unit (see Table 3). In many cases, the dye load was estimated as a so-called “owf” value, or as grams of dye taken for dyeing “over the weight of fabric”. Sometimes this corresponds to a dying solution from which all the dye has been expectedly absorbed into the tissue. Indeed, this is not always the case, even though such a calculation can be considered valid as a first approximation. More accurately, the amount of adsorbed dye was calculated by measuring the absorbance of the dyeing solution before and after application to the fabric. Looking at the data collected in the Table 3, most of the dyes were applied in the range of 0.1–3%. Sometimes the amount was as low as 0.02–0.4% [33,52,60,61], or as high as 9–14% [30,49]. The surface concentrations of the dyes ranged from µg/cm^2^ to mg/cm^2^. The surface concentrations, however, can only be properly compared when the density of the carrier textile is known. Considering the density of cotton as being between 100 g/m^2^ (fine shirt) and 300 g/m^2^ (chino trousers), a 150 g/m^2^ value can be taken as a good estimate. In this case, 0.02 mg/cm^2^ of the dye **11** [47] would correspond to 0.13%, 76 µg/cm^2^ of the dye **48** [64] to 0.5%, and 0.4 mg/cm^2^ of the dye **2** [45] to 2.6%. Vice versa, the above-mentioned range of 0.1–3% for a 150 g/m^2^ cotton would mean dye loads of 0.15–4.5 g/m^2^. For dyes **3** and **20**, which were effective under indoor light (see Table 2), the loads were 0.016% and 0.15%, correspondingly [16,18].

Generally speaking, a larger amount of PS yields stronger inactivation. However, the influence has a complex character as the photodynamic action takes place in a porous fibrous material both inside and at the surface. Because of its complexity, this aspect is rarely considered in detail, although some articles studied the influence of the chromophore’s load [7,17,18,19,23,28,31,33,58]. In most of these cases, the efficacy increased with an increased amount of PS, but the dependence was not linear. In the work of Flores et al., the 1% PS concentration was more efficient than the 3%, probably because of the aggregation and screening of the dye molecules [7]. Studies of aggregation were also performed by Efimov et al. [16].

The morphology of the pristine and dyed fabric was most commonly studied by SEM, and in one case by AFM [25]. Typically, the imaging revealed no significant structural difference between the modified and unmodified textile, and the layer of a dye is basically not possible to detect. However, when the dye is combined with a polysiloxane or other polymer matrix, the results can be visible on SEM imaging [42,43,46].

The mechanical properties of the textiles before and after modification were tested in a few articles [12,20,27,39,50,51,54]. The tests revealed that the dyeing process does not affect the mechanical strength of the textile or decreases it slightly. Similarly, the thermal stability of the fabrics was tested in most of the articles with the TGA method and once with DSC [32]. The studies revealed that the dyeing process usually slightly reduces the thermal stability of textiles, but they remain stable up to 300 °C. Rub fastness was assessed for copper phthalocyanine, Rose Bengal, and silkworm excrement extracts **17**, **33** [12], **29** [54], and **53** [35].

The light fastness of the PATXs and their ability to maintain their antimicrobial activity upon prolonged exposure to light was studied only by a few groups. Jiang et al. noticed that the activity of tetracationic porphyrin 9 reduced by 10 times after 2 h of irradiation [46]. Wright et al. reported that the combination of tetraphenylpoprhyrin and Rose Bengal (compound 13) bleaches out under disinfection conditions [27]. Tetracaboxylic porphyrin in MOF (compound 15) was stable at least for 30 min [50]. Similar stability was demonstrated for phthalocyanines covalently appended to a polymer support 16 [20]. Zinc phthalocyanine 17 exhibited a 1-log decrease in efficiency after 2 h of irradiation and a 2-log decrease after 4 h of exposure to light [51]. Similar stability was observed for Rose Bengal 29 [54] and its combination with an indolinium dye (compound 33) [51]. Dyes from the set 28 [39] and pigments of the silkworm excrement 53 (most probably chlorophyll derivatives) [35] demonstrated a 1-log decrease in activity after 12 h of exposure to light. Tetracationic zinc phthalocynaine 20 was stable after 6 h of illumination at 36,000 lux without any loss of activity [16]. Triphenilamine derivative 48 remained stable and active after 14 days of illumination under office light [64].

The wash fastness of the dyes was tested in a few different ways. For the compounds 2–7, 9, 10, 15, and 16, the fabric was washed with various solvents after synthesis, but no additional tests of the leaching or activity changes were performed [3,18,19,20,30,33,41,43,45,46,49]. Catsriciano et al. studied the release of porphyrin 11 from the matric fabric [47].

Combined tests of washing and activity were performed by a few groups. Nie et al. revealed that depending on the composition of the fabric, porphyrin 15 may withstand from 1–2 up to 10 washes with a 1-log decrease in activity [48,50]. Similarly, anthraquinone-based dyes, Rose Bengal and indolinium derivatives, and silkworm excrement chromophores 24, 26, 27, 28, 53 demonstrated 1–2-log decreases in activity after 1–10 wash cycles. Phthalocyanine 20 retained its activity after five washing cycles [16]. Wash fastness was thoroughly tested according to AATCC 107-2017 [75] and AATCC 61-1996 [76] for compound 48 by Li et al., and no reduction in activity was observed after 100 wash cycles [64].

### 2.6. Conclusions

The health system is constantly affected by infectious complications, termed nosocomial infections, which constitute a serious problem for public health worldwide. Bacteria are the main causes of NIs, which result in up to $4.5 billion in additional healthcare expenses annually. Infectious diseases are responsible for 10 million deaths, representing almost 20% of all fatalities worldwide, and it is estimated that approximately 80% of the human infections occur from microbe-contaminated surfaces. The traditional approach to eradicating bacteria is mainly antibiotic drugs, which are not very efficient because of the development of effective resistant mechanisms that bacteria use to survive. In addition, the limited penetration of drugs, from both membrane and biofilm structures, results in reduced susceptibility to this kind of treatment. Additionally, products from the textile industry, especially those made from natural fibers, provide an excellent environment for growth of microorganisms due to their large surface area, ability to retain humidity, and capability to keep oxygen, heat, and nutrients. Most textile materials used in hospitals and hotels are cross-infection vectors or transmitters of diseases caused by microorganisms. Hence, the design of self-sterilizing materials with photoinduced antibacterial and/or antiviral activity is important for different applications, ranging from materials for medical and clinical practices to the disinfection of objects for public use. There is a huge unmet demand for self-disinfecting textiles and filters, which would be equally effective against current and, most importantly, future pandemics.

This review shows that the photodynamic principle is a very efficient technique to eradicate different microorganisms, such as Gram-positive and Gram-negative bacteria, viruses, and fungi. Illumination by visible light with a photosensitizer in the presence of molecular oxygen generates reactive oxygen species, most notably singlet oxygen (^1^O_2_), can lead to very efficient antibacterial or antiviral activity.

This review also shows that there is great diversity in the textiles, photosensitizers, and light sources being used. The lack of standardization in (i) the measurement of light doses and (ii) the determination of antimicrobial or antiviral activity is certainly the main factor limiting the spread of these textiles. What is more, very few studies have examined the influence of ageing and leaching on these textiles and their antimicrobial activity. Similarly, the long-term influence of illumination on the efficacy of these textiles has not been addressed almost at all, even though test methods exist in the literature.

It would therefore be desirable for the various researchers involved in the development of photoantimicrobial and photoantiviral textiles to organize themselves, perhaps through an academic society, in order to propose standardized measurement and control protocols for these textiles and to publish articles from which the obtained results could be compared. Such scientific rigor could also be useful to convince the industry and government bodies of the value of these innovative textiles.

## Figures and Tables

**Table 1:** Data on the photoinactivation of bacteria, fungi, and viruses. The numbers in the left part of the table indicate the achieved logarithm of inactivation of a pathogen (in the column title) by the compound (in the row name), i.e “6” is 99.9999% inactivation and 0.1 is 40% inactivation.

Compound	Ref.	*Staphylococcus aureus*	*Pseudomonas aeruginosa*	*Escherichia coli*	*B. subtilis*	MRSA Methicillin-Resistant *S. aureus*	*S. epidermidis*	*M. smegmatis*	*E. faecalis*	*B. cereus*	*K. pneumoniae*	*Aspergillus fumigatus*	*C. albicans*	Influenza	SARS-CoV 227	Non-Enveloped Feline Calicivirus	Autographa Californica Multiple Nuclear Polyhedrosis Virus (AcMNPV),	MHV-A59	Standard	Light	Time, min	Light Dose, J/cm^2^	Mode
**2**	[45]	5																			1440	9.5	surface
**3**	[18]	4		3				4														0.1	suspension
**4**	[19]	2		2																			surface
**5**	[3]													4						30,000 lux	240	432	surface
**6**	[41]	4		0.2																		9.5	surface
**7**	[43]	n/d			n/d															1440 lm 15 W LED			Agar + fibers
**8**	[42]											1								15,500 lux	180		Fabric + liquid
**9**	[46]	5							5		6					3				65/80 mW/cm^2^	60	288	surface
**10**	[33]	0.5	0.2	0.1–0.3																White light 0.36 mW/cm^2^	90	1.9	surface
**11**	[47]	4	0																AATCC100	50 W Halogen	10/30	5	surface
**22**	[24]	2		1.3																365 nm, 4 W or 8 W	120		surface
**12**	[40]																1.3			300 W	30		surface
**13**	[27]			1		1									1					530 nm	120		surface
**14**	[28]	0.5		1																Hg 100 W, 43 mW/cm^2^	30	77	surface
**15**	[49]	6	6	6	6															500 W Xe 15 cm	30	56	surface
**15**	[30]	6	6	6	6															500 W Xe 15 cm	45	56	surface
**15**	[50]	6		6																500 W Xe 15 cm	30	56	surface
**15**	[48]	6		6																500W Xe 15 cm, 31.45 W/cm^2^	30	56	surface
**16**	[20]	2		2		2													AATCC100	150 mW/cm^2^	10	90	surface
**17**	[51]	3		3		3													AATCC100	660 nm, 75mW/cm^2^	10	45	surface
**19**	[23]	1–4																		UVA light			surface
**20**	[16]														4				ISO 18184	LED, 590 lux	30	1	surface
**21**	[52]																			635 nm, 36 mW	60		surface
**22**	[24]																			365 nm, 4 W or 8 W	120		surface
**23**	[4]	2		2																			surface
**24**	[11]			3		3														sunlight-driven			surface
**25**	[53]			3																350 or 420 nm			Fabric + liquid
**26**	[34]			1.3–6															AATCC100	1380 lm 50 W 15cm			surface
**27**	[17]			3																500 W Xe, 420 nm	60		surface
**28**	[39]	4		4											4					500 W Xe 65 mW/cm^2^	60	234	surface
**29**	[7]								5											6.75 mW/cm^2^	20	8	Fabric + liquid
**29**	[55]	5				4														Xe 35 mW/cm^2^	60	126	surface
**30**	[56]											6								16,000 lux	300	288	surface
**30**	[8]												0.2–0.3						ASTM E2149-01 [73]	15,500 lux	180	167	Fabric + liquid
**31**	[9]												0.2–0.3						ASTM E2149-01	15,500 lux	180	167	Fabric + liquid
**32**	[26]			3		3														520 nm	30	60	Fabric + liquid
**33**	[12]	4			4															500 W Xe 12 cm	60		surface
**34**	[13]			3																Xe 500 W, 12 cm	60		surface
**35**	[32]	6		6																Xe lamp, 5 mW/cm^2^	60	18	surface
**36**	[31]	3																		500 W Xe 20 cm	30		Fabric + liquid
**37**	[56]																			520 nm–25 mW			surface
**38**	[57]		1							1										HL 8325, 25 W, 1230 lm	60		surface
**39**	[58]		3																	sunlight	60		surface
**40**	[59]									1										HL 8325, 25 W, 1230 lm	60		surface
**41**	[60]	1.5																		sunlight	1080	648	Fabric + liquid
**42**	[61]																			sunlight	1080	648	Fabric + liquid
**43**	[6]	2		2																Xe 500 W, 12 cm	30		surface
**44**	[10]	4		4															AATCC 183-2014 [74]	Simulated daylight	5	200	surface
**45**	[38]			0.5–1	0.1–0.2															Sunlight	360	216	surface, Agar
**46**	[62]	1		1																808 nm, 2.0 W/cm^2^ and 660nm	600	1200	surface
**47**	[63]	4	2	4									1.7							White light 45 mW/cm^2^	20	54	surface
**48**	[64]																	5		White light 3 mW/cm^2^	8	1.44	surface
**49**	[65]	4																		Xe 500 W, 12 cm	60		surface
**50**	[66]	3		3																808 nm, 300 mW/cm^2^	300	90	surface
**51**	[5]						4													White light, 9.5 mW/cm^2^	60	34	surface
**52**	[25]	2–3																	ISO 22196	LED 50 W, 470 nm, 50 cm	60		surface
**53**	[35]					4														Xe lamp 60,000/8000/800 lux			surface

**Table 2:** Approximate light doses for different illumination conditions.

Lighting Conditions	lux	mW/cm^2^	Time	Light Dose J/cm^2^	Light Dose Range
Direct sunlight	100,000	100	30 min	180	100–1000
Bright day	10,000	10	30 min	18	10–100
Shadow outdoors	1000	1	30 min	1,8	1–10
Indoors	500	0,5	30 min	0,9	0.1–1

**Table 3:** Summary of the dye loads and stability tests. For the light fastness and wash fastness columns, the logs of loss of antimicrobial activity are given where available.

Comp	Ref.	Dye Load	Dye Load-Activity	Morphology	Mechanical Properties	Rub-Fastness	Thermal Stability	Light-Fastness	Washing Stability
**1**	[44]								
**2**	[45]	0.4–0.6 mg/cm^2^							washed
**3**	[18]	0.16 umol/mg	tested				TGA, 320°C		washed
**4**	[19]	3.9% owf	tested	SEM			TGA, 300 °C		washed
**5**	[3]			SEM					washed
**6**	[41]	1.5%		SEM			TGA, 300 °C		washed
**7**	[43]	12%		SEM					washed
**8**	[42]	20–30%		SEM			TGA, 300 °C		
**9**	[46]	0.4–5 ug/mg		SEM			TGA, 300 °C	1 log loss in 2 h	washed
**10**	[33]	0.003–0.3%	tested	SEM			TGA, 300 °C		washed
**11**	[47]	0.02 mg/cm^2^		SEM					release studied
**12**	[21]								
**12**	[40]	1%		SEM					
**13**	[27]	1.5%		SEM	tested			bleaches out	
**14**	[28]	0.1%	tested	SEM					
**15**	[30]	9.1%		SEM					washed
**15**	[48]			SEM					1–2 log loss in 5–10 wash cycles
**15**	[49]	14%		SEM/TEM					washed
**15**	[50]	0.4 mg/cm^2^		SEM	tested		TGA, 300 °C	30 min	1 log loss in 1–2 washes
**16**	[20]	0.2% owf		SEM	tested		TGA, 300 °C	30 min	washed
**17**	[51]	3%		SEM	tested	tested	TGA	1–2 log loss in 2–4 h	tested
**18**	[29]								
**19**	[23]	0.5–8%	tested					4 cycles	
**20**	[16]	0.15%						24 h stable	5 washes stable
**21**	[52]	0.04%							
**22**	[24]			SEM			TGA	2 h UV	
**23**	[4]	rechargeable		SEM			TGA		
**24**	[11]			SEM					0–5–2 log loss 1–5 washes,
**25**	[53]								
**26**	[34]	0.2–9.6 umol/g		SEM			TGA, 300 °C		48 whash cycles
**27**	[17]	2.5%	tested	SEM			TGA, 300 °C	2 log loss for AQ in 7 days	3 log for AQ in 3 wash cycles
**28**	[39]	0.23–0.33%		SEM	tested		TGA, 270°C	1–2 logs loss in 12 h	1–2 log loss upon washing
**29**	[7]	0.5, 1, 3% owf	tested						
**29**	[54]			SEM	tested	tested	TGA	2 logs loss in 5 h	
**29**	[55]	30–80 umol/L		SEM					
**30**	[8]			SEM					
**30**	[56]								
**31**	[9]			SEM					
**32**	[26]								
**33**	[12]	1–3%		SEM	tested	tested		1–2 logs loss in 2–4 h	tested
**34**	[13]	1%							
**35**	[32]			SEM			DSC		
**36**	[31]	1–3%	tested						
**38**	[57]	0.17–0.19%							
**39**	[58]	0.02%	tested						
**40**	[59]	0.5–1%		SEM					
**41**	[60]	0.02%							
**42**	[61]	0.02%							
**43**	[6]			SEM					
**44**	[10]			SEM			TGA		
**45**	[38]	24–36 mg/cm^2^		SEM					
**46**	[62]			SEM					
**47**	[63]	0.05–0.3%		SEM					wash stable
**48**	[64]	43–76 ug/cm^2^		SEM				5–14 days stable	100 washes stable
**49**	[65]	33%		SEM					
**50**	[66]			SEM					
**51**	[5]			SEM					
**52**	[25]	2%		AFM					
**53**	[35]	1%				tested		1–2 log loss in 12 h	1–2 log loss in 7 washes
